# Does Juvenile Play Programme the Equine Musculoskeletal System?

**DOI:** 10.3390/ani9090646

**Published:** 2019-09-03

**Authors:** Chris W. Rogers, Keren E. Dittmer

**Affiliations:** 1 School of Veterinary Science, Massey University, Private Bag 11 222, Palmerston North 4410, New Zealand; 2 School of Agriculture and Environment, Massey University, Private Bag 11 222, Palmerston North 4410, New Zealand

**Keywords:** horse, bone, play, foal, mechanostat

## Abstract

**Simple Summary:**

Locomotor play is a common behaviour expressed across a diverse range of species. As a cursorial animal, the horse is capable of locomotor activity within a relatively short time after birth. In the foal, spontaneous locomotor play occurs early in life and has an obvious role in the development of locomotor skills. The intensity and vigour of locomotor play increases with age and this, in turn, provides cumulative increases in the loads the musculoskeletal system experiences. These progressive cumulative loading cycles (bouts of locomotor play), in both the timing and magnitude, reflect the microstrain required to stimulate bone development based on the mechanostat theorem. Data from the published literature were presented to provide empirical support for this hypothesis. Thus, spontaneous locomotor play may be vital to ensure optimal bone development in the horse. Modern production systems need to provide appropriate opportunities for foals to perform spontaneous locomotor play to optimise bone development and reduce the risk of future musculoskeletal injury later in life.

**Abstract:**

In mammals, play behaviour appears innate and, because of this, may provide insight into the frequency and intensity of load that is required to stimulate positive musculoskeletal development. The objective of this review was to explore the interaction between play and tissue (bone) development at a molecular through to whole-animal level, with specific focus on the horse as a model. The basis of our understanding of the response of bone to loading is the mechanostat theorem. This assumes that at a tissue level, bone attempts to keep localised strain within the physiological range of 1500–2500 microstrain. Loads above this range result in a modelling response to reduce strain, and strain below this threshold results in remodelling to maintain the localised physiological range. In foals, locomotor play is dramatic and vigorous, with cumulative increases in both intensity and complexity. Based on published literature describing locomotor play in foals and the microstrain at different gaits in the horse, it was proposed that locomotor play in foal aligns with the mechanostat theorem in both the magnitude and frequency of load cycles applied. The cumulative increases in the complexity and intensity of locomotor play as the foal develops, in turn, ensure the strain rates associated with play remain above the local physiological range and promote material and architectural changes in the distal limb bones. Thus, spontaneous locomotor play may be vital to ensure optimal bone development in the horse. Modern management systems need to provide appropriate opportunities for foals to perform spontaneous locomotor play to optimise bone development and reduce the risk of future musculoskeletal injury later in life.

## 1. Introduction

The Barker hypothesis, or the developmental origins of health and disease (DOHAD), provides a framework in which we can explain the plasticity and developmental potential of tissues and organs and their subsequent response to environmental stimuli [1]. Fundamental to this theorem is the proposal that there are sensitive periods in the development of the foetus, and neonate, which determine the developmental path of the tissue, organs and even the individual. These changes in developmental potential have been reported to have intergenerational persistence. Such changes optimise the individual for the environment in which it will live. In modern society, the horse is kept for athletic purposes, predominantly sport and racing. Within these production systems, the largest reason for involuntary loss is musculoskeletal injury. The DOHAD implies that strategic manipulation of the early foetal or neonatal environment could have a positive effect on musculoskeletal development, and thus reduce the involuntary losses associated with musculoskeletal injury. However, before this can occur, we need to understand how the foals’ early life experiences could influence musculoskeletal development.

### 1.1. The Precocious Neonate

**Figure 1:**
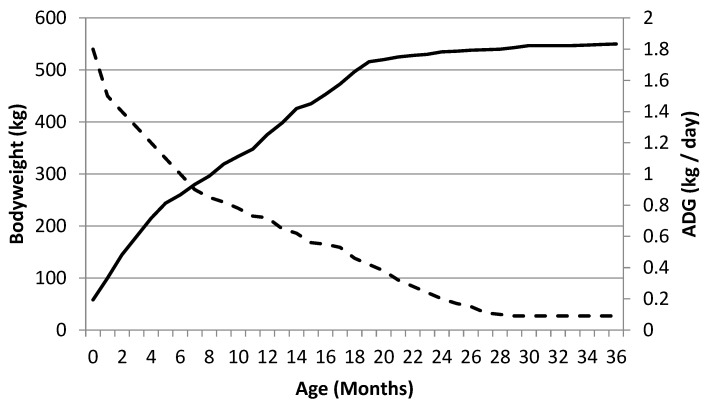
Bodyweight and average daily gain for a Thoroughbred from birth to 36 months old (- - - - body weight (kg), **―** ADG (kg/day)).

The ecological niche occupied by the horse has shaped a cursorial animal that is capable of covering large distances at low to moderate speeds and high-speed bursts of acceleration to avoid predation. This adaptation for locomotor efficiency is not restricted to the adult, but can also be observed in the foal, even as a neonate. The foal is capable of standing and suckling quickly after birth and is capable of reasonably coordinated locomotor activity within the first few hours of birth. This rate of rapid development continues for the first year of life, with rapid increases in both bodyweight and wither height. In the thoroughbred, it is reported that a 50-kg foal will have an average daily gain of over 1.5 kg/day until weaning (~6 months) and then continue to grow at ~0.7 kg/day until 14 months when it will have achieved ~90% of mature height (Figure 1) [2].

Across mammalian species, play behaviour is an integral part of the developmental process of the individual. In the context of human development, play has been recognised to have an integral role in the development of attributes as diverse as priming the musculoskeletal and neuromuscular processes, and the development of social and cultural skills. From an evolutionary perspective, the primary driver of play activity in the foal appears to be priming the musculoskeletal system for future challenges, both in the short term and later in life as an adult.

### 1.2. Innate Play in the Foal—What Does It Do?

In both feral and most commercial management environments, locomotor play provides the obvious stimulus for modification and focus of the rapid growth and development. Play behaviour appears innate and, because of this, may provide insight into the frequency and intensity of load that is required to stimulate positive musculoskeletal development [3,4]. The objective of this review was to explore the interaction between play and tissue (bone) development, at a molecular through to whole-animal level, with specific focus on the horse as a model.

## 2. Bone Adaption

Bone is a dynamic tissue that changes both its shape and material properties in response to strain or load. This phenomenon has long been recognised and was first described by the German anatomist Julius Wolff in the 19th century. Wolff’s law states that “every change in the function of a bone is followed by certain definite changes in its’ internal architecture and its’ external conformation”. A refinement of this observation was the application of the mechanostat theory, which was able to describe the response of either bone deposition or resorption directly to the magnitude of strain to which the tissue was exposed [5]. Across species, the minimum effective strain (MES) for bone appears constant, with bone altering architecture and material properties to ensure that the localised strain is within what appears to be the ideal physiological range of 1500–2500 microstrain (μE).

Experimental studies have demonstrated a “dose response” effect on bone, with strains below the ideal physiological range inducing bone resorption and decreases in bone cross sectional area. Conversely, strains above the threshold increased bone mass and cross-sectional area [6]. The magnitude of the response was proportional not just to the size of the load, but also the frequency of loading, with a maximal response observed at 36 cycles at 0.5 Hz per day. Maintenance of bone mass required fewer load cycles, in this case as few as four cycles per day [6].

Based on the mechanostat theory, the adaptive mechanism of bone to respond dynamically to load and the concept of a sensitive period to prime tissue, Sinclair et al. [7] proposed, “horses that are used for competitive events that include movements outside the natural range on a frequent basis should be introduced to such exercise at an early age when architecture of the skeleton could be influenced to adapt to the loading demands”. In relation to ontogeny, this implies that locomotor play may be not only a positive stimuli but also an essential stimuli to ensure correct musculoskeletal development.

### Bone Cells, Mechanosensation and Transduction

Recent evidence suggests that the bone cell primarily responsible for sensing and responding to mechanical stimuli is the osteocyte [8]. Osteocytes arise from osteoblasts that become encased in mineralised osteoid during bone formation [9]. Despite this entrapment, they connect to other osteocytes and cells on the surface by means of branching cytoplasmic processes that traverse via canaliculi and form the lacunar-canalicular system (LCS). The LCS is responsible for the detection and conversion of mechanical strain into changes in bone formation and resorption [10]. Cyclic bone compression results in load-induced fluid and solute flow through the spaces between the osteocyte cell body and processes, and the lacunar-canaliculi walls [11,12,13,14]. The osteocyte cytoplasmic processes are tethered to projections on the canaliculi wall by proteoglycans (in particular perlecan) and integrins of the pericellular matrix; when fluid moves past these attachments, this is thought to create drag forces that allow amplification of the strain to the level required to obtain a biological response [11,15].

A number of different structures have been shown to be involved in mechanosensation and mechanotransduction, including primary cilia, ion channels (voltage sensitive calcium channels), gap junctions (in particular connexin 43, pannexin 1), and integrins (α1 and α3 integrins) [16,17]. Regardless of the type of mechanosensor, the initial response to fluid shear forces is thought to result in a release of ATP from vesicles within osteocytes, the level of which depends on the amount of mechanical stimulation [18,19]. ATP then activates purinergic receptors (ATP gated cation channels), leading to increased intracellular calcium concentrations [18] that are thought to be responsible for increased nitric oxide [8,20] and increased expression of cyclo-oxygenase 2 (COX2) leading to increased PGE2 [21].

Increased nitric oxide and PGE2 lead to activation of the cAMP/protein kinase A (PKA), Akt/phosphatidylinositol 3 kinase (Akt/PI3K), focal adhesion kinase (FAK), mitogen activated protein kinase (MAPK), extracellular signal-related kinase 1/2 (ERK1/2) and wnt-β catenin pathways [22,23,24,25]. Ultimately, the activation of these pathways results in decreased osteocyte apoptosis, decreased sclerostin expression resulting in increased bone formation, and decreased RANKL: OPG ratio resulting in decreased osteoclast differentiation and osteoclastic bone resorption [25,26,27,28].

Studies examining the compression and tension sides of teeth have shown that strains in the range of 1500–3000 microstrain result in decreased RANKL and increased osteoprotegerin (OPG), suggesting that there is decreased osteoclastic resorption [29,30]. However, strains greater than 3000 microstrain are associated with microdamage. Unfortunately, there are few data on MES and mechanostat theory from a cellular perspective. Many scientific publications have examined the effects of different types of strain on osteocytes, osteoblasts and monocyte/macrophage cell lines; however, the strains required to obtain a response in cell culture far exceed those exerted on bone in situ, making direct comparison of strains challenging. This is an active area of research, whereby models of osteocytes in a 3-dimensional native or imitation bone matrix are being developed [31].

Nonetheless, as a general rule at a cellular level, when modelling, low strain leads to apoptosis of osteocytes and decreased bone mass by removal of bone from the endocortical surface of the cortex and from trabeculae [32]. Normal strain results in the maintenance of bone mass, and high strain results in an increase in bone mass by deposition of bone on existing trabeculae and periosteally in the cortex. Similarly, during remodelling, low strain leads to increased bone resorption, normal strain maintenance of bone mass, and increased strain results in microdamage and targeted remodelling to replace damaged bone [32].

## 3. Play

Energy conservation and the allocation of resources to activities is an important concept in the ontogeny of the horse. In a life history framework, the allocation of resources (energy) to play is often considered secondary to growth and brain development [33,34]. In primates, this trade-off has been clearly demonstrated [35], but the same has also been demonstrated in a diverse group of other species [36]. In horses, possibly due to their ecological niche as a cursorial animal, this observation is not so clear, though it appears that maternal condition, and hence, energy available for the neonate, may be positively associated with increased play behaviour in male foals [4]. Possibly due to the energetic cost/trade-off with play behaviour, it has been reported that play most often occurs in periods of ontogenic flexibility and thus, provides the greatest return on the energy expended [37]. The timing in a life history framework of play behaviour may also be dependent on the “motor training hypothesis” where physical play accelerates motor skills acquisition, which, in turn may enable higher levels of play (in terms of physical load and complexity), generating a compounding effect on the animals’ developmental potential.

Despite the economic and cultural importance of the horse, the literature describing play and locomotor play under commercial management systems is relatively sparse. Until relatively recently, most data on play behaviour in foals relied on observational data. The first ethogram for foal play behaviour was described by McDonnel & Poulin [38]. Using this ethogram, Cameron et al. [4] described play behaviour in feral horses and identified that greater play behaviour was shown by foals with dams that lost more condition (i.e., had a greater energetic investment in the foal). Greatest frequencies of play were observed in male foals, and the authors proposed that the timings of the greatest frequency of play bouts coincided with developmental periods that may maximise or encourage development of the foals’ musculoskeletal system. Support for this hypothesis was provided in the data demonstrating that the foals involved in the most play had better survival and body condition as yearlings [4].

**Figure 2:**
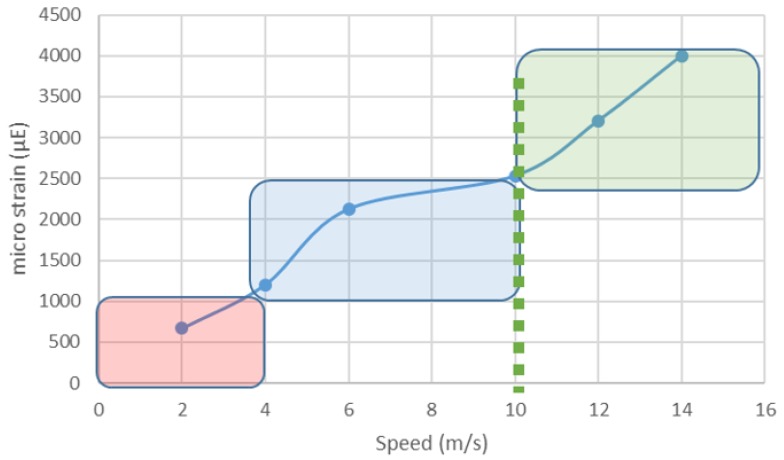
The relationship between velocity (m/s) and micro strain (µE) on the dorsal surface of the third metacarpal bone (MCIII). Loads in the in the red region are below the MES, blue represents the MES, and green represents loads above the MES. Data derived from [5,42].

In feral environments, GPS tracking of (brumby) horses in Australia has demonstrated that they routinely travel 8–28 km/day, predominantly at the walk whilst grazing or browsing [39,40]. Under commercial management conditions, (i.e., mares and foals at pasture) foals are able to cover ~7 km/day within the first three days of life. When resources are sufficient (adequate feed supply and paddock size not limiting movement), this 7 km/day appears to be the norm in domestic conditions and would provide a moderate load at the lower end of the peak strain rate scale (~600 µε) (Figure 2). Therefore, this strain would be unlikely to have a significant osteogenic effect [41].

In contrast, play is characterised by short (not prolonged) bursts of high speed activity, the ideal recipe to provide the micro strain rate to stimulate the musculoskeletal system [42,43]. From an ontogeny perspective, it appears that the greatest bouts of play may be early in the developmental life of the foal. Using GPS and observational data, it was reported that foals spent 1.09 ± 2.88% of the time in canter (including play—bucking, rearing, mounting) when aged 0–4 weeks old [44]. This value was three times the quantities observed in recordings up to 16 weeks old. Translation of this data to a daily workload load index (velocity * distance) demonstrated that the workload index of the foals in the first 4 weeks of life was approximately twice that of the following 3 months. This peak activity early in the first month of life has also been reported from observational studies of Camargue horse foals [45].

Two factors may contribute to the greater levels of osteogenic exercise observed in the first month of life. The first is that the primary source of nutrition during the first month is mares’ milk and this may negate the need for time-consuming activities such as grazing, meaning that there is more time and energy resources to allocate to play [45]. The other relates directly to the ontogeny theory and the application of load at a time (age) when the tissue is most receptive, so that maximal response or developmental potential can be achieved. At present, the data from the examination of cartilage development from foals provided with additional exercise and restrictive exercise early in life provides the strongest support for the theory that there is a sensitive period for tissue priming. The turnover rate of the collagen network of the extracellular matrix of articular cartilage decreases to almost nil at the time of maturity. The application of an exercise load prior to weaning, either in the form of pasture exercise vs. loose box confinement, or additional canter exercise to foals at pasture, demonstrated a stimulatory effect (increased glycosaminoglycan component and greater chondrocyte viability) that persisted to later in life [46,47,48,49].

The same early exercise provided a stimulatory effect on bone. Localised increases in volumetric BMD in the third tarsal bone and the third carpal bone appeared transitory in the 5 month vs. 11 month model [50]. With the provision of additional canter exercise to pasture-kept foals (~30% increase in workload) there was a significant increase in both bone size and strength in the first phalanx and the third metacarpal bone, which became quantifiable at 12 months of age [51] and persisted during and after race training [52]. The delay in identifying changes in bone, even though additional exercise was provided from 3 weeks of age, within the proposed sensitive period, may relate to the methodologies used to prospectively measure changes in bone content and morphology (pQCT) and that much of the early response may have been focused on architectural changes rather than gross morphology. Evidence for this is provided by an elegant loaded and unloaded mouse femur study, where the architectural potential of the unloaded femur failed to occur [53]. Thus, loading is required to achieve architectural refinement and optimisation of the tissue. Without exposure to a sufficient range of loads through play, it is probable that bone not only lacks architectural refinement but has not developed appropriately to have the future potential to tolerate and respond to the maximal or upper limit of the physiological range. This development may not be so important from an architectural perspective, but rather in defining the bounds for the mechanostat so that there is establishment of the feedback loop for limited or no response to the occasional abnormal strain.

The effect of exercise on muscle development should also be considered, as muscle contractions provide the low magnitude, high-frequency dynamic strain considered most effective at producing bone formation as per the mechanostat model [54,55]. Bone mineral density and bone strength are strongly correlated with muscle development in children, and to a lesser extent in adults [56]. Together, bone and muscle form the so-called bone-muscle unit, and at a genetic level, there is considerable cross talk between skeletal muscle and bone. Skeletal muscle may produce insulin-like growth factor-1 (IGF-1), basic fibroblast growth factor 2, interleukin-6, osteoglycin and osteoactivin, all of which have an effect on bone [57]. Conversely, in bone, osteocytes produce PGE_2_, Wnt3a and sclerostin, osteoblasts produce osteocalcin and IGF-1, and chondrocytes produce Dickkopf-1 and Indian hedgehog, all of which may have an effect on skeletal muscle cells [57].

### 3.1. Play and the Mechanostat Theory

Based on the data of Rubin & Lanyon [6], a positive bone response would be expected with > 2000 ± 30 µE applied at 0.5 Hz for 36 cycles, with a loading of 4 cycles per day, to maintain the response. How does this data fit with the loading of the distal limb of the foal during bouts of play during the first month of life?

The stride frequency of the horse operates within a relatively narrow range and in foals, could be averaged to ~0.6 s per stride, which equates to a loading rate of ~1.5 Hz [58]. In fact, the kinematic pattern of gait is highly conserved with ontogeny and has given rise to the concept of horses having a kinematic fingerprint. This in itself may be a developmental process to conserve the rate and direction of loading that the foals’ limb experiences with growth and subsequently throughout life.

In mature horses, data indicates that the MES is exceeded during canter at 8–10 m/s [42]. At the canter, the greatest strain (acceleration/deceleration) on the distal limb occurs at the initiation of stance, with rapid deceleration of the limb [59], and at the initiation of propulsion, with the application of muscle and tendon forces [60]. These strains are exaggerated in locomotor play with short bursts of rapid acceleration and deceleration on turns [59,61]. Locomotor play may also include large strain outside the sagittal plane, which may also be important for priming the tissue of the distal limb for the excessive/abnormal strain associated with a misstep, and thus aid in injury prevention.

### 3.2. Play Should Be Osteo-Inductive

Using these data and the data from Kurvers et al. [44] we can calculate the number of potential osteo-inductive load cycles applied in early life. If canter activity was expressed as 1.09% of all daily activity, then this equates to 15.7 min of canter/play per day or 39 s/h, which is similar to estimates provided by for feral horses [4]. With a stride frequency of 1.5 Hz [58] this translates to a daily load of 1,412 load cycles per day in the first 4 weeks, dropping down to 466 load cycles per day in weeks 9–16. These total daily loads exceed the 36 cycles required for bone response. Thus, it may be the application of sequential bouts of play that are maximising bone response and development.

**Figure 3:**
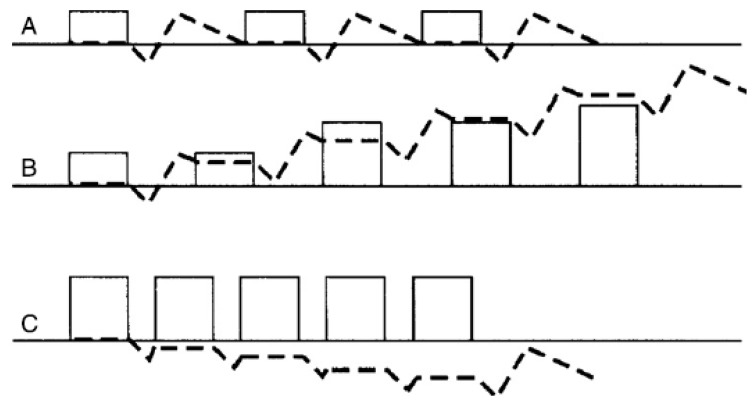
Diagrammatic representation of load (boxes) and bone response (dotted line) for (**A**) lack of response due to sufficient load but too long a recovery period between loads, (**B**) the expected pattern associated with bouts of play with increasing response in relation to an increasing load and appropriate recovery period, (**C**) overload due to insufficient recovery period (Adapted from [62]).

Based on the mechanostat theory and experimental data, cumulative cycles of load, with rest periods, result in a greater osteo-inductive response. This response cycle fits the theorem of load and response that is applied in sport science to describe the appropriate application of load to maximise the response of tissue, presented in Figure 3 [62].

Bouts of individual gallop play in foals are generally short 0.19 ± 0.01 min (~11 s or 19 load cycles) and greater in bouts of group galloping 0.42 ± 0.04 min (~25 s or 42 load cycles) [63]. Both of these activities in young foals would be high impact. Individual gallop play is characterised by circles of 2–3-m diameter and rapid acceleration and deceleration. Bouts of group galloping are also characterised by rapid acceleration and deceleration [45,63].

Using the data derived earlier [44], the foal is exposed to approximately 1412 osteo-inductive load cycles (high impact steps). If 36 consecutive loads are required by the mechanostat, then the foal could theoretically generate approximately 39 loading sessions at a rate of 1.6 bouts/loading sessions per hour. These estimates may be an underestimate of the rate of loading as the assumption was made of even distribution over a 24 h period. Data from an observational study of welsh ponies reported that galloping and high speed play bouts were observed 3.6 ± 0.9 times per hours in the first 4 weeks of a foal’s life [63]. As foals mature, there is an increasing complexity and vigour to the play activity, which in turn, may provide a greater cumulative osteoinductive effect.

### 3.3. In Modern Management Systems, Play Drives Orientation of the Tissue for Future Load

These data indicate that play provides not just an immediate effect on bone development but may also stimulate the identification of the biological thresholds for load and stimulate the direction of bone development and architecture. Our ability to understand the establishment of biological thresholds and macro and micro architecture has been limited by our imaging modalities, and that quantification of these changes has required serial termination experiments [64]. There are, however, hints within the existing literature that support the hypothesis that early exercise/play primes the musculoskeletal system. Early work by a team at Utrecht University demonstrated that restricted exercise (confinement to a stall management system) resulted in the blank joint—whereby cartilage within the metacarpophalangeal joint – articulating surfaces of distal third metacarpus and proximal first phalanx) lacked biochemical heterogeneity. In contrast, foals kept at pasture, able to exercise freely and demonstrate play behaviour had cartilage at the same site with heterogeneity and variation, reflecting the variation in sites of loading during a range of velocities (standing through to gallop) [41,65]. This ability to express natural play behaviour was not associated with gross changes in bone morphology but did reduce the prevalence of OC lesions, demonstrating the subtle interaction of early exercise and tissue development [50].

In a subsequent study using young thoroughbreds, similar findings were obtained when additional exercise (~30% increase in workload) was superimposed on top of pasture reared foals [2]. The additional (high speed) exercise increased chondrocyte viability in the cartilage of the distal MC3, demonstrating a tissue response to the applied load [49]. However, significant changes in bone morphology and structure, measured using pQCT, were not able to be quantified until the age when the horses entered race training (~2 years old), though these changes did persist during the subsequent racing career [66].

**Figure 4:**
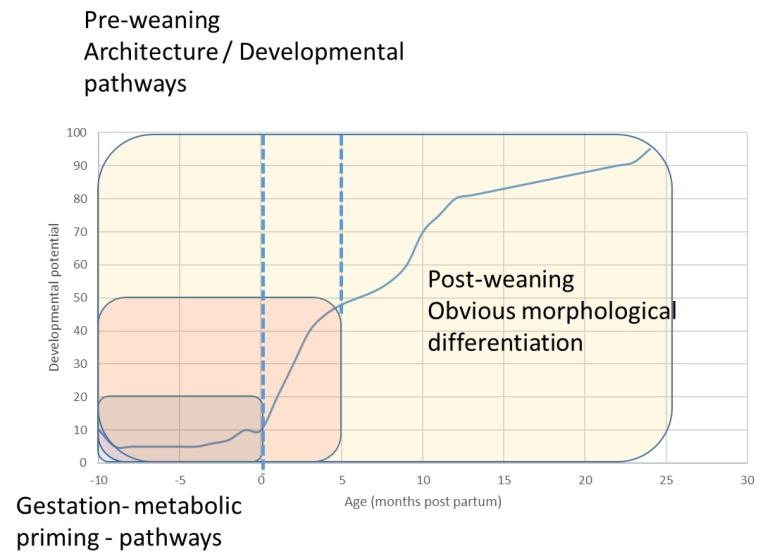
Conceptual framework for bone development and sensitive periods for response in the equid.

These data demonstrate that in a cursorial animal with rapid early growth potential, such as the horse, the effect of bouts of high-speed play behaviour may not be overtly obvious with our current imaging modalities. The primary purpose of play is to establish the physiological bounds of activity, and hence, loading that the distal limb is exposed to and to refine the developmental path of the structural orientation and development of bone tissue. This concept is presented visually in Figure 4.

## 4. Conclusions

This review attempted to draw together the concept of the mechanostat, the cellular response of bone and how play activity in foals may provide the ideal cumulative levels of strain to optimise bone development. In conjunction with published data from other tissues (most notably cartilage), it appears that play behaviour in foals not only maps the immediate response of bone but provides the developmental framework for future response. The increasing vigour and complexity of play as the foal matures reinforces the concept of cumulative adaptation and the necessity to provide appropriate stimuli early in life to capitalize on this cumulative compounding effect.
